# Advances in Ultra-High Performance Concretes and Cementitious Composites

**DOI:** 10.3390/ma19081543

**Published:** 2026-04-13

**Authors:** Jialiang Wang, Baoguo Han

**Affiliations:** 1 Department of Civil and Architectural Engineering, Aarhus University, 8000C Aarhus, Denmark; 2 School of Civil Engineering, Dalian University of Technology, Dalian 116024, China

## 1. Introduction

Ultra-high-performance concretes (UHPCs) and advanced cementitious composites have emerged as a key class of materials for next-generation infrastructure [1] due to their superior mechanical performance, enhanced durability, and increasing potential for sustainability [2,3,4,5,6]. However, due to the demands of modern construction, which include a longer service life, higher resilience, reduced maintenance, and lower environmental impact [7,8,9], the development of high-performance cement-based materials has shifted from purely strength-driven optimization toward a more integrated consideration of durability, functionality, environmental friendliness, and energy and resource efficiency [10,11,12,13,14,15,16,17,18,19].

To support such developments, we launched the Special Issue “Advances in Ultra-High-Performance Concretes and Cementitious Composites,” which provides a platform for recent advances in material design, durability, sustainability, structural applications, and modeling approaches. The collected contributions reflect the multidisciplinary nature of this field, bridging materials science, structural engineering, and environmental performance evaluation.

## 2. Overview of Published Contributions

This Special Issue comprises six papers, including one review and five original research articles, covering a broad spectrum of topics from material innovation to structural applications.

Focusing on material innovation and sustainability, Liu et al. [20] investigated the incorporation of phosphogypsum into UHPC matrices, demonstrating its effectiveness in enhancing mechanical performance while reducing shrinkage and thus offering a promising pathway for industrial by-product utilization. In parallel, Yuan et al. [21] combined experimental and numerical approaches to study the mechanical behavior and damage evolution of cemented tailings backfill, contributing to sustainable material development and resource recycling.

In terms of durability and environmental performance, Qi et al. [22] examined the salt-frost resistance of high-performance concrete reinforced with polypropylene fibers, highlighting its applicability in harsh service environments such as cold and marine regions. Meanwhile, Chen et al. [23] investigated the influence of relative humidity on the creep behavior of cement paste, providing valuable insights into time-dependent deformation mechanisms under environmental coupling conditions.

Regarding structural applications, Fernández et al. [24] evaluated the use of thin high-performance fiber-reinforced concrete (HPFRC) jackets for strengthening axially loaded reinforced concrete columns, demonstrating the effectiveness of advanced cementitious materials in structural retrofitting and performance enhancement.

Finally, a comprehensive review by Ignatkhina and Moustafa [25] assessed the feasibility of UHPC for nuclear waste storage applications, focusing on durability, thermal behavior, and nuclear performance requirements. This work highlights the potential of UHPC in highly demanding and safety-critical infrastructure systems.

Overall, the contributions collectively cover key aspects outlined in the initial scope of the Special Issue, including material design, durability, sustainability, structural applications, and experimental–numerical analysis. However, topics such as advanced processing technologies and unified modeling frameworks remain relatively underrepresented, indicating potential directions for future research.

## 3. Key Insights and Emerging Trends

The studies included in this Special Issue reveal several important trends in the development of UHPC and advanced cementitious composites.

First, there is a clear transition from conventional strength-oriented material design toward performance-integrated design, where durability under complex environmental conditions and sustainability-driven material modification are simultaneously emphasized. The incorporation of industrial by-products such as phosphogypsum and the exploration of tailings-based systems reflect a growing shift toward resource-efficient cementitious materials.

Second, environment–material interactions have become a central research focus. Investigations on salt-frost damage and humidity-dependent creep behavior demonstrate that long-term performance is strongly governed by coupled environmental factors, rather than intrinsic material properties alone. This highlights the necessity of considering real service conditions in material design and evaluation.

Third, there is an increasing trend toward multi-scale understanding achieved through combined experimental and numerical approaches. The integration of laboratory testing with modeling enables a more comprehensive interpretation of mechanical behavior and damage evolution, bridging the gap between microstructural mechanisms and macroscopic performance.

Finally, UHPC is becoming increasingly prominent due to its potential for high-value and specialized applications, including structural strengthening and nuclear infrastructure. These applications impose stricter requirements on durability, reliability, and long-term stability, further driving the evolution of cementitious materials from conventional construction materials toward engineered functional systems.

## 4. Challenges and Future Perspectives

Despite the progress highlighted in this Special Issue, several ongoing challenges remain. Firstly, it is necessary to achieve a robust balance between high performance and sustainability, particularly with regard to reducing clinker content while maintaining durability and long-term stability. Existing strategies, although promising, are often system-specific and lack generalizable design frameworks.

In addition, the prediction of long-term performance under coupled environmental conditions remains limited. The complexity arising from interactions among microstructure evolution, moisture transport, and external loading requires the development of multi-scale and multi-physics modeling approaches.

Another important gap is the lack of unified design methodologies linking material composition, microstructural characteristics, and structural performance. Future research should aim to establish more systematic frameworks that enable predictive and programmable material design.

Emerging directions may include the development of low-carbon UHPC systems, advanced in situ characterization techniques, and data-driven modeling approaches. Furthermore, the design of materials with adaptive, self-responsive, or self-healing capabilities may provide new opportunities to enhance the resilience and service life of infrastructure.

## 5. Conclusions and Acknowledgments

This Special Issue provides a timely overview of recent advances in ultra-high-performance concretes and cementitious composites, addressing material innovation, durability, sustainability, structural applications, and emerging technologies. The collected works highlight both the scientific progress and the practical relevance of these materials in addressing contemporary engineering challenges.

The Guest Editors would like to express their sincere gratitude to all authors for their valuable contributions and to the reviewers for their rigorous and constructive feedback. We also thank the editorial staff of *Materials* for their continuous support throughout the publication process. We hope that this Special Issue will serve as a valuable reference and stimulate further research and innovation in the field of advanced cementitious materials.
